# A Qualitative Examination of the Impact of the COVID-19 Pandemic on Individuals with Neuro-developmental Disabilities and their Families

**DOI:** 10.1007/s10826-022-02336-8

**Published:** 2022-07-14

**Authors:** David B. Nicholas, Wendy Mitchell, Jill Ciesielski, Arisha Khan, Lucyna Lach

**Affiliations:** 1 Faculty of Social Work, Central and Northern Alberta Region, University of Calgary, 3-250, 10230 Jasper Avenue, Edmonton, Alberta T5J 4P6 Canada; 2 Centre for Research on Children and Families, McGill University, Suite 106, Wilson Hall, 3506 University Street, Montreal Quebec, H3A 2A7 Canada; 3 School of Social Work, Faculty of Arts, McGill University, Suite 300, Wilson Hall, 3506 University Street, Montreal Quebec, H3A 2A7 Canada

**Keywords:** Neuro-developmental disabilities, Families, COVID-19 pandemic, Social determinants of health, Service disruption, Qualitative

## Abstract

Individuals with neuro-developmental disabilities (NDD) have been profoundly affected by the COVID-19 pandemic. Based on focus groups with 24 service providers supporting this population, using an Interpretive Description approach, we examined perceived impacts of the pandemic on individuals with NDD and their families. The results highlight pandemic-related experiences which include: service reduction, the need for financial supports, relying on natural supports, and school-related challenges. Interruptions in services have resulted in intensified mental health issues for individuals with NDD and family caregivers, with particular concern for those with added social determinants of health-related barriers. Mitigating factors have also emerged, such as resilience and technology utilization to facilitate communication. Recommendations for resource flexibility and sufficiency as well as navigational support are offered.

## Highlights

- Focus groups were conducted with 24 service providers to learn about the impacts of the pandemic on individuals with NDD and their families.
- The COVID-19 pandemic has been difficult for individuals with neuro-developmental disabilities (NDD) who required supports, and for their families.
- Many community supports and services for this population were reduced during the pandemic, leading to difficulties, confusion, and outrage for individuals and families.
- Stress and caregiver burnout were described, with additional struggles faced by those with additional social determinants of health barriers.
- Generative factors were also described, including family resiliency and the leveraging of technology.

Neurodevelopmental disabilities (NDD) are brain-based disorders that are characterized by effects on “cognition, communication, behaviour, and/or motor skills” (Mullin et al., 2013); these disabilities include autism spectrum disorder, fetal alcohol spectrum disorder, cerebral palsy, attention deficit hyperactivity disorder, intellectual disability, communication disorders, learning disorder, and motor disorder (American Psychiatric Association, 2013). Based on studies examining worldwide pandemics, there is evidence to suggest that the secondary stressors from the COVID-19 pandemic will escalate among individuals with NDD both during and after the pandemic (Koller et al., 2006; Maunder et al. 2003; Nicholas et al., 2020a; Sprang & Silman, 2013). There has been a substantial and protracted reduction of developmental, health and social care, such as services delivered by physical, occupational, and speech and language therapies (Trabacca & Russo, 2020), along with extended disruptions in schooling and employment (Salt et al., 2020; Statistics Canada, 2020a). The absence of guidelines in these areas has left health and social care providers to grapple with uncertainty and complex programmatic and ethical quagmires and disparities (LeBlanc et al., 2020; Russo & Trabacca, 2020; Schiariti, 2020; Trabacca & Russo, 2020).

Research has shown that adverse childhood experiences can have life-long impacts on the developing child’s physical and mental health (Committee on Psychosocial Aspects of Child and Family Health et al., 2012; Sprang & Silman, 2013). How systemic shifts and personal stresses will emerge from the COVID-19 pandemic has yet to be seen. The disruption of daily routines and separation from caregivers/support personnel have been shown to heighten risk for disrupted adjustment and growth, including mental health concerns and developmental regression (Cassidy et al., 2020; Fung & Ricci, 2020; Schonfeld et al., 2015). Past research has also found that pandemic-related shifts have resulted in strain in care and emotional impact for affected individuals and their families (Koller et al., 2006).

## Pandemic-Related Shifts and Their Impacts

Clinicians and researchers have noted substantial societal and service changes for persons with disabilities and mental health issues as a result of the pandemic. In some cases, services have temporarily ended or been shifted according to safety protocols such as modified or online delivery (Fung & Ricci, 2020; Somekh et al., 2020). In terms of impacts of such service changes, some literature suggests the potential for relief among youth with anxiety or other mental health concerns as some stressors, such as school and social-based demands, may have lessened due to pandemic restrictions (Nicholas et al., 2020a). On the other hand, worries, family strain, and mental health challenges have been heightened, and are anticipated to escalate as society transitions back from social isolation to ‘normal’ life (Nicholas et al., 2020a).

While the use of technology in health and social care has seen vast uptake during the pandemic, service providers have reported both benefits and concerns with online-mediated services. Increased navigational access to specialized services for those in rural settings can be offered, yet online services pose challenges due to modality differences such as potentially limited or more opaque self-presentation and communication (e.g., decreased non-verbal cues) in computer-based exchange (Nicholas et al., 2020a). Additionally, technology has not yet advanced to the point of replacing many aspects of clinical examinations that rely on face-to-face procedures, leaving providers concerned about the ability to gather key metrics or validly conduct assessment and intervention. On the other hand, in-person visits in the pandemic may provoke anxiety due to the required use of personal protective equipment (PPE), such as face masks, particularly among some children with NDD and/or mental health issues (Nicholas et al., 2020a).

The pandemic has resulted in additional negative impacts on populations who already experience marginalization. Among children with mental health and behavioral needs, an American commentary noted that 80% of these children rely on school-based services (Masonbrink & Hurley, 2020). The closure of schools has come in tandem with a loss of vital resources for children/youth with disabilities and/or other vulnerabilities. Further, the lack of in-home support to assist with virtual learning and behavioral and emotional needs, likely has burdened parents who may be insufficiently equipped to facilitate remote learning and needed therapies, and/or who already may be experiencing heightened stress and challenge due to pandemic shifts (without adding on this additional layer of demand). Furthermore, individuals with disabilities are disproportionately represented in Canadian statistics of poverty; disability can be both a cause of poverty, but is also amplified by poverty (Council of Canadians with Disabilities, 2013). As an example of the effects felt on another marginalized group, the pandemic has disproportionately impacted women in the workforce. Canadian data has found that women accounted for more employment losses then men at an average of 53.7% of the total losses, despite making up 47.3% of the pre-COVID total workforce (Grekou & Lu, 2021). Mothers in an American study reported taking time off or altogether leaving employment due to safety concerns, but also because of school closures, a lack of childcare options, and increased home, work and caregiving responsibilities (Ranji et al., 2021).

Special education and other developmental supports for students with NDD often require physical contact and redirection, interpersonal prompting, and close attention to the motivational structure of the environment. Further, those with sensory or other impairments who require assistive technology that is usually available in school settings, may not have access to these resources in home-based learning (Masonbrink & Hurley, 2020). In-person schooling has resumed in some jurisdictions, yet concerns remain about insufficient use of PPE and physical distancing in classrooms, with risk to safety. Accordingly, individuals with disabilities have faced, and continue to face, challenges adapting to the shifting environment, and those who require comprehensive supports generally remain substantially under-served (Government of Ontario, 2020).

A recently published survey by a coalition of Canadian autism organizations demonstrated the significant impact of the COVID-19 pandemic on the autism community (Salt et al., 2020). Less than a fifth of the respondents reported coping “well” or “very well” during the pandemic. Mental health was rated as a primary concern, reflecting disruption in activities of daily living and difficulty accessing supports and services. A survey that used crowdsourcing to obtain its sample was conducted by Statistics Canada (Arim et al., 2020). In that survey, 1 in 5 respondents identified having a child between the ages of 0 and 14 years with a disability. Just over three quarters (76%) of parents of these children were very or extremely concerned about managing their children’s behaviours, stress, anxiety and emotions, in contrast to 57% of parents of children without disabilities. A higher proportion of parents of children with disabilities were very or extremely concerned about the extent of their children’s screen time, loneliness or isolation, mental health, and school/academic functioning.

Resource gaps that have been amplified in the pandemic appear to reflect pre-existing service gaps and a lack of prioritization of resources in society for disabled people. These gaps warrant scrutiny as they reflect ableism that marginalize disabled populations (Nicholas et al, 2020b; Mitchell & Snyder, 2015). Such realities may have been amplified and/or made more visible in the pandemic, but have continually existed. Rendering these gaps and oppression visible and redressing them at structural, community, and service levels, need to be priorities in moving forward. This invites critical research that elicits, and reflects upon, lived experience, in the aim of societal transparency and social justice. To address these aims and better understand pandemic-related experience, we elicited the effects of the COVID-19 pandemic on individuals with NDD and their families, from the vantage point of service providers supporting them. We opted to seek perspectives from service providers due to sample convenience and because service providers’ practices engaged many diverse individuals with NDD and families. Our hope was that consideration of the vast diversity of individuals and families would be represented through exploring the perceptions of those who engage with, and provide support to, this population. As part of a larger study, specific research questions addressed in this paper are:

1. What have been the experiences of individuals with NDD and their families as they have navigated the COVID-19 pandemic?
2. What have been the impacts of service reductions and shifts during the pandemic?
3. How, if at all, have individuals with additional social determinant of health barriers been differentially impacted by the pandemic?

## Methods

An Interpretive Description (ID) qualitative inquiry approach (Thorne, 2016) methodologically guided this study in its aim of amplifying the experiences of children, youth and young adults (to approximately 24 years) and their families. ID is ideal in its focus on identifying ‘on the ground’ experiential findings in the aim of practically informing practice and care planning. Emerging from nursing, ID offers discursive and analytic techniques to identify stakeholder experiences, perspectives, patterns and interpretations to inform practice and programming (Thorne et al., 2004). The practical nature of this approach in linking experiential data to practice gain and social justice, justified its used in this study.

Participant recruitment emerged from study publicity by agencies offering health, mental health, education and social services to this population We further recruited participants via snowball sampling. Detailed information about the study was conveyed to potential participants, and if interested in participation, informed consent was obtained from each participant. Participants were informed that their engagement in the study was voluntary and confidential. Institutional review board approval was received by the University of Calgary Conjoint Faculties Research Ethics Board prior to study commencement, and participant anonymity was maintained.

Focus groups were guided by a semi-structured focus group schedule which posed open-ended questions about individual and family experiences that service providers have encountered. The use of focus groups was helpful and expeditious in deeply and rapidly engaging service providers in a comprehensive dialogue about the impact of the pandemic on families as well as service implications. Focus groups entailed questions of participants such as: (1) How has the COVID-19 pandemic impacted individuals with NDD and their families?, and (2) What, if any, are the impacts of practice shifts resulting from the pandemic on individuals with NDD and families? Conducted by authors DBN and WM, the focus groups were convened between June and October, 2020.

Five focus groups were facilitated via Zoom technology, with approximately 5 individuals per group. Focus groups comprised a single session for each participant, lasting a range of 60 to 90 min. They were audio recorded, and subjected to transcription and inductive line-by-line coding, categorization and thematic organization (Corbin & Strauss, 2014), with the support of NVivo 11 qualitative data management and analysis software (QSR International, 2018). The data were reviewed by a coder (JC), with a proportion of the data also reviewed and analyzed by two other team members (WM, DBN) who concurred on coding consistency and consensus on emerging codes. Findings were corroborated based on peer-debriefing, inter-rater reliability, referential adequacy within the presentation of results herein, and presentation to families who validated the resonance of themes, thus member checking.

Practicality of information for practice and policy advancement, based on an ID-oriented orientation to data elicitation for guiding practice, was attempted in situating findings in ways that inform practice, programming and policy. Accordingly, data analysis as well as the presentation of findings have been oriented to practical service quality improvement in the context of pandemic conditions.

## The Sample

Twenty-four interdisciplinary service providers across a variety of fields including respite and support workers and professionals in the fields of psychology, occupational therapy, and speech-language pathology, participated in focus groups; 23 of whom were female, and one was male. Participants ranged in years of experience in the field from 2.5 to 45 years, and in 4 cases, participants had additional lived experience as a parent of a son/daughter with NDD. Service providers resided and worked in one of the sampled provincial/territorial regions of Canada: Yukon (*n* = 13), Alberta (*n* = 6) and British Columbia (*n* = 5). These regions span urban and rural communities, and all service providers offered services during the pandemic, including counselling, respite services, early education, after-school programming, family support, developmental and professional services, navigational support, school and vocational support services, and administration/leadership. Populations served (and thus represented herein) primarily comprised individuals (largely children and youth as well as, in some cases, adults) with autism, fetal alcohol spectrum disorder or cerebral palsy, and their families.

## Results

Participants consistently conveyed how difficult the pandemic had been for individuals with NDD who required resource and/or intervention support, and for their families. They expressed concern that individuals with NDD had lost previously-accessed services due to service closures and social distancing requirements. Families/family caregivers have had to adjust their goals and priorities, as illustrated by a service provider who also is the mother of children with NDD: “My goal has changed from having my kids to be the most successful that they can be to having them not kill each other each day.” Participants offered many examples of reaching out for services, yet receiving insufficient help. Findings emerged in three broad domains: (A) pandemic shifts, (B) impacts on individuals and families, and (C) generative or mitigating factors. In the first domain of pandemic shifts, as addressed below, we will convey experiences of service cessation or modification, financial need, reliance on natural supports, and educational issues for children/youth. This is followed by an exploration of impacts, including intersectional challenges for NDD populations particularly with co-existing vulnerabilities. Finally, generative factors that have been mitigative even amidst the prevailing adversity of the pandemic, consist of family resilience and the leveraging of technology for community support. These experiential elements are briefly conveyed below, with corroborating quotes from the data.

### Pandemic-Imposed Shifts: Service Cessation

Participants stated that services to individuals with NDD have been reduced, including school, developmental services, health and mental health resources, recreation, and other care/support in the community and home. They described extensive difficulties for individuals and families, and a range of confusion and outrage due to the shuttering of services including government offices, as illustrated by a participant:

That is just absolutely ridiculous and so the system needs to be much more responsive because they’re just downloading, using the pandemic and not providing the services they said they were going to provide. It’s… appalling that group homes or other services don’t see themselves as essential services. This individual’s mental health complexity doesn’t go away… I think people have accountability and responsibility, and they need to own what they own, and be respectful to the needs of (individuals with NDD)… during this unending, uncertain length of a pandemic.

Participants objected to processes that ultimately determined *essential* versus *non-essential* services. A participant described her concern about what she viewed to be idiosyncratic means of determining the fate i.e., closure, of services for people with disabilities. She stated,

People do not consider [service to people with developmental disability] to be an essential service… [But] if you can get the right supports in place, it minimizes secondary disabilities as well as we talk about quality of life. Those [services] are really important. It may not be the same as diagnosing cancer and getting surgery right away. But I really think we have to look at disabilities with that lens.

Many participants expressed worry that a lack of access to agency intervention and social support would negatively and longitudinally affect those with NDD, with particular concern about mid and longer-term impacts. They noted that the current severe lack of supports in the disability community – already insufficient prior to the pandemic – is now strained unacceptably and unsustainably, particularly for those with complex care needs and their families:

At the beginning, people accepted things that needed to be sacrificed for the sake of the greater good. But it gets difficult to sustain that over a longer period of time. Within six weeks or two months, that’s when we really saw… families with high needs really struggling and families in crisis.

Participants expressed concern about the lack of mental health services particularly as mental health struggles were inflamed by pandemic-related worry and imposed restrictions. A participant noted that in some cases, service providers were not even answering their phone or responding to messages. In some cases, individuals had appointments for mental health supports after being on a waitlist for a significant period of time, but those appointments had been cancelled due to the pandemic. In other instances, in-home services were offered, but families were not comfortable with an agency’s COVID-19 safety protocols. And if families were in quarantine, they reportedly were not able to access supports at all, which was difficult for families, especially when the individual was experiencing severe behavioral challenges, and/or when parents lacked other supports (e.g., some single parents, parents without an informal network of support). One parent caring for a youth with NDD was described to have struggled to access help for her son’s aggressive behaviors, which the participant feared would, without intervention, result in others in the household getting hurt. Another participant stated that at the government ministry level, individuals and families with support needs were directed to contact provincial social workers for support, but social workers were not receiving direction on how to respond to these needs. These social workers therefore were unsure how to respond, with some reportedly not answering phone requests. Another participant noted that some youth/adults with NDD and parents rely on being able to visit their social worker or agency office to ask a question or seek support. These individuals, they suggested, often did not easily adjust to having to contact service providers by telephone or online. Another participant felt that the government had not been sufficiently clear to the public about reopening guidelines. Even with some easing of pandemic restrictions over time, participants noted a continued lack of services and communication.

#### Financial support need

Many individuals and families were reported to experience financial struggles due to lost income and/or unemployment as well as ‘out of pocket’ expense to address basic care needs. Further, participants reported discrepancies whereby some individuals/families have received funding without asking for it, and others have not qualified despite desperately needing the assistance. Those whose taxes were not up-to-date could not access financial supports, but they also could not receive help to complete their tax forms. Individuals in one jurisdiction who received government funding for specialized developmental supports were not only reported to be unable to access these service providers during the lockdown, but also were not able to use their funding for anything else. Similarly, those who were approved for respite funding but who could not access respite due to pandemic restrictions, were restricted from pivoting these funds for any other purpose despite a myriad of urgent needs.

Participants noted that funding flexibility would be helpful during the pandemic. Moreover, given the lack of available services, many families, by necessity due to the lack of alternative service providers, assumed extraordinary levels of additional complex care and/or homeschooling roles, and in some cases, had to stop work to care for the individual with NDD, without additional support – financial or otherwise. Some were paying out of pocket and, in specific cases, taking on debt to access a needed service that was not covered. Several stated that available information about emergency funding was unclear, and families were unsure if they were eligible. Further, the criteria for eligibility changed frequently such that even professional navigators who sought to help families admittedly struggled in helping clients navigate funding.

#### Reliance on natural supports

By necessity, many individuals and family caregivers relied on their natural supports such as extended family members more frequently than before the pandemic due to the lack of available professional or para-professional support during the pandemic. One participant emphasized the importance of building natural support networks: “Post-pandemic, you need to build your natural supports. You need to nurture your natural supports so that when supports are pulled, you have a foundation of support.” Yet, it was noted by participants that many individuals and families lacked a strong natural or informal support system and rather relied on professional supports. Accordingly, such a recommendation to nurture natural supports may not be feasible and unfairly place the onus on families to provide specialized and necessary support.

Moreover, several participants noted that reliance on natural supports such as aging parents or grandparents to help with caregiving could heighten risk for COVID-19 contagion, with particular concern about the vulnerability of populations such as seniors and those with co-existing conditions.

#### Educational issues

Participants shared that virtual education for children/youth with NDD generally has been of poor quality and a negative experience for many of these students. Many families were described as feeling abandoned by the education system as schools reportedly had not done enough to support students with NDD. Government cutbacks in school services, reportedly occurring in one jurisdiction during the pandemic, resulted in additional losses of supports such as aides, which then placed even more pressure on teachers and parents. Some parents received an overwhelming amount of daily instruction and requirements from schools and school districts, but reportedly not enough information about the future in terms of what to expect in moving forward. Service providers reflected on their own and parents’ worries about how difficult and overwhelming the transition back to school in-person may be particularly for youth with co-existing mental health issues, and they were variably unsure about how to optimally prepare youth for that eventuality.

In contrast, participants also acknowledged a variety of positive experiences in which some children/youth have been doing better academically through virtual education. In particular, some with ASD or ADHD have benefitted from learning in an environment with fewer distractions and demands. A service provider who also is a parent of a child with NDD, shared that she had been able to communicate with, and teach, her child in a more effective way than had school personnel. Overall in these data, there were discrepancies in school-related experiences among families because some had received more support from teachers and educational assistants than others. Yet in each case, this required extensive involvement by parents who had varying levels of time as well as ‘special education’ skills, which in turn reportedly has influenced child/youth educational outcomes. In such cases, parents buffered the immense impact of the strained school system for their child with NDD.

### Impacts on Families

The pandemic has affected individuals and families deeply as they have grappled with multiple concerns about personal and family vulnerability in the face of seeking well-being. For some, developmental support lapsed, with concern about interrupted advancement. Impacts on various members of the families, as well broader impacts on communities, are conveyed below.

#### Impacts on children with NDD

Participants observed that since the pandemic began, many children with NDD have become more anxious, some reportedly picking up on their parents’ stress (and vice versa). Children were described to verbally or behaviorally express frustration and anger, and in some cases, have difficulty understanding the situation. Like their peers, many reportedly struggled with not having contact with others (e.g., friends, grandparents). Participants were concerned about the long-term effects that isolation and the lack of supports would have, especially on children with co-existing mental health issues. This was reported to be exacerbated by service reductions: “I think it’s a perfect storm. We have all these issues that are going to collide and are colliding; loss of jobs, loss of an aide, loss of school supports, loss of funding… I mean, it’s all going to accumulate and… will disenfranchise this generation of children.”

#### Impacts on youth and young adults with NDD

Participants indicated that youth and adults with NDD also have been profoundly impacted by COVID-19 and its associated challenges. Settings such as group homes and other congregate living facilities have had higher rates of COVID-19 and substantial resource insufficiencies. Day programs have been closed or, if continuing, programming has been severely diminished. Lacking daily structure and social isolation, many individuals with autism were described as particularly struggling with the drastic change in routines due to pandemic restrictions.

It was observed that service reduction was unequally challenging for neurodivergent adults; some of whom are more transient and many of whom are more socially marginalized than neurotypical peers. One participant noted struggles for this group in navigating the judicial system (e.g., court, probation), and connecting with landlords for youth/young adults experiencing housing insecurity or vulnerability. Another participant stated that some youth and young adults with NDD were not staying at home because they did not fully understand pandemic guidelines, or their routine and social connections were largely outside of their home. She noted that for some:

Their whole day is centered around leaving their home in the morning and finding their social circle in town all day, and then maybe winding their way home at the end of the day, and that involved a connection with the soup kitchen and perhaps a connection with mental wellness supports. You can’t just tell those people to go home or stay home when their whole life doesn’t revolve around anything to do with their house except sleeping. And so that is very challenging. People don’t understand sometimes there’s a group of 10 people that hang out on the church steps and really that’s their family. Despite the fact that they don’t all sleep in the same place, they are a family. Those people maybe aren’t going to keep two meters apart from each other because that’s their bubble; those bubbles look different in our community.

#### Impact on parents/family caregivers

Parents/family caregivers experienced negative impacts such as isolation, feeling overwhelmed, and experiencing anxiety as a result of the pandemic. A participant illustrated such challenges and emotions:

With the care needs being placed on the families, there’s a lot of caregiver burnout. I’ve heard of family caregivers who went to the ER because they were having physical clinical pains and it turns out it wasn’t that, it was an actual mental breakdown and the mother had to be admitted into the psychiatric ward. So of course that creates a whole new dynamic. I think for a lot of families it is overwhelming.

Similarly, another participant noted with concern:

Families [are] struggling so much that they’re considering putting their children in care (relinquishing caregiving to the state) because they can’t care for their child, and that has been highlighted through this pandemic that families can’t do this alone, and when they’re forced to do this on their own, things crumble and they crumble big time.

Participants described extensive pressure currently being exerted on parents/family caregivers to provide greater levels of supervision and hands-on complex care because such services had been reduced or ceased. Families simultaneously were dealing with uncertainty in not knowing when supports would resume, or what services will look like upon resumption. Several participants of children who required complex care, identified parents who feel hesitant to, and in some cases, afraid to reach out for help. For instance, a parent of a youth with NDD reportedly shared:

When I reached out to our social worker to say that we were really, really struggling with everybody’s mental health and those outbursts and whatnot, our social worker said to me, ‘Should I call protection in to come and do an investigation on your family to make sure that everybody’s safe?’ At that moment, I was reaching out for help, and when I heard that it scared me to my core.

With the service provider’s response in this instance, seeking help was immediately ceased due to the parent’s experience of a rupture in trust of the very system that is mandated to help families. At such a vulnerable point for this family resulting from strains associated with the pandemic, attempts to ask for needed service were abandoned due to a perceived threat to family safety.

Beyond overall family impact, the stress of the pandemic reportedly had different impacts on various family members. Mothers had largely been left with additional NDD care responsibilities along with other demands of work and care in the pandemic (e.g., working from home, home-based schooling support, care for aging parents). Nuanced and challenging paternal impacts were also noted. As an example, participants described fathers whom they felt had been uniquely impacted due to widespread layoffs in the primary industry of their region. These fathers reportedly had lost their job and source of income, *and* assumed new and extensive at-home care responsibilities for their child with NDD about which many were previously largely unfamiliar. Parents further were described as having to weigh the different needs of all their children, and were variably struggling with concerns that non-disabled children, also with pandemic-related and other needs, may be missing out on parental attention due to extraordinary NDD-focused family care that was heightened during the pandemic.

#### Challenges for NDD populations with other vulnerabilities

Many participants reported an increase in issues such as drug and alcohol use, domestic violence, mental health exacerbation, suicide ideation, and poverty due to higher levels of stress, financial strain and job loss, and lack of supports. When crises arose, service providers were called upon, but often did not respond as they had prior to the pandemic as services have been shuttered or reduced. A few participants thought that the influx of money through emergency government funding to individuals – without any support for handing it in daily life amidst deep personal struggles – had resulted in increased substance use among vulnerable groups. Tragic instances of resulting suicidality and violence were noted.

In response to struggles facing the marginalized population of individuals with NDD and substance use, one remote community offered small amounts of substances to individuals with addictions as a harm reduction approach when the only local liquor store in the region closed due to the pandemic. This community response sought to prevent withdrawal among individuals in recognizing that if such withdrawal happened across this cohort of the community, the local health and social care system could be overwhelmed. Such an example illustrates the ingenuity yet vulnerability of health and social care systems in communities during the pandemic.

Participants expressed food insecurity to have become an even greater challenge for some individuals with NDD and co-existing poverty. One rural community’s food bank and soup kitchen closed due to the pandemic, and the service community had to find an alternative to ensure basic sustenance. In that case, participants felt that the pandemic vicariously has brought greater attention to, and awareness of, crises associated with indicators of inequity, and the resultant disproportionate deleterious impacts of the pandemic on already vulnerable NDD populations.

Affordable housing shortages is another community and societal issue that reportedly has been amplified by the pandemic. Participants indicated that finding housing has become much more challenging during the pandemic particularly for individuals/families affected by NDD and who have been evicted, are hard to house, or otherwise are in transition. Furthermore, with many social service agencies and NDD-related services closed or reduced, more vulnerable individuals with co-occurring complexities have struggled to address basic functional needs such as access to restrooms and hygiene, the Internet, medication, and required paperwork for income assistance. Transportation also has been a barrier, particularly in rural and remote communities. And in some urban centers, public transportation that was free or subsidized early in the pandemic is often no longer subsidized as it was. Many rural communities lack public transportation or a taxi service, and rely on ridesharing or hitchhiking to access services. Such practice is highly risky at best, but variably prohibited during the pandemic due to physical distancing requisites. Institutionally, many organizations have directed their employees to not transport clients as they had prior to the pandemic. Accordingly, some individuals with NDD in those communities have found it even more difficult to attend appointments or access services (if available). During warmer Canadian summer months, people were able to walk to more places, but this has been made more difficult in the colder winter weather of northern climates, and walking to resources is further limited in rural locations due to long distances.

One rural community shut down most in-person services and was operating with only one outreach worker for the entire region, resulting in unmet service needs. Inconsistency of rules guiding service delivery has further impeded clarity about what is available and how to access what is available. As an example, service providers in a jurisdiction reported being instructed to not provide transportation to probation appointments, but could meet with their clients, lend them their phone to call a probation officer, and then confirm to the probation officer over the phone the client’s identity. Such procedurally complex, detailed and shifting sequalae and restrictions, especially when differentially applied across various organizations, imposed cumulative layers of confusion in terms of what and how services were accessible.

### Generative Factors: Family Resilience

Despite pervasive system and service gaps, some individuals and families experienced an increased sense of connection as a result of spending more time together as a family due to pandemic impacts that have kept families at home. A participant stated:

Families (with a child/youth with NDD) have become so involved with their loved one. They are right up there doing the art, dancing around, humming along, doing the craft, answering the questions. I haven’t seen moms and dads and caregivers do that before. This has given them [time and opportunity], and families say, ‘we didn’t know that our child loved that much art, we didn’t know they could tap their fingers to a song’. And these parents are learning much more about their child because… we send our child every day – since they were in kindergarten – off to a program… We don’t see them, and when we see them, we are caregiving… we are not *experiencing* them [as we are now].

Another related benefit is that some families experienced fewer imposed demands from their typical schedules and appointments, which opened up time and opportunity for engagement, as illustrated below in a rural and northern setting:

Like now it’s hunting season, I know lots of families are out hunting right now whereas last year, it was kind of a sporadic type of thing. But now it’s more like, ‘Okay, we’re gathering all of our family members and we’re going out to the bush, and we’re spending a week or two at the cabin and really getting back out on the land’. So I think it’s given people the opportunity to do that again.

In some cases and to varying degrees, individuals and families became more resolute in recognizing that they know what is best for them in their circumstances, and embraced the importance of not letting the system dictate what will work for them/their family. Reflecting on this resolve, a participant recounted an individual who said, “Yeah, I’ve got this. I know what’s best for my family”.

In encouraging these shifts, accessible support from service providers and advocates was viewed as helpful. This entailed encouraging individual and family well-being, but also, “giving [individuals and families the message]: ‘if you reach a brick wall or a barrier, we are so accessible, we are a phone call away, an email, so you’re not alone in this’.”

The importance of self-care and establishing individual/family routine was noted. One participant concluded that individuals and families directly impacted by NDD, were used to adapting because they continually had to adjust to challenging circumstances and resource shortages and issues. Yet it was noted that some families were managing well while others were having great difficulty managing amidst the existing gaps. Among individuals and families in general, however, they reportedly have had to be even more flexible which, to varying degrees, has imposed strain and challenge.

#### Leveraging technology for community support

Participants expressed relief and, in some cases, surprise, in noting that some service recipients successfully pivoted to virtual programming. Despite limitations, online services were noted to provide a necessary connection for some who were more isolated. One participant described technology-based communication to be a “lifeline” in the pandemic, and another stated that some clients needed to just talk to someone, even if they did not need anything specific. Multiple participants noted that some clients actually preferred virtual programming and supports. While certainly not the preference of all families, a participant estimated that 20-25% of their clients (children/youth with NDD) indicated a preference for virtual service delivery.

This shift to online services was described as particularly helpful for families who lived a distance from treatment centers. Some with NDD reportedly also found programs at home to be less overwhelming due to not being required to attend appointments in a strange or distant environment. Facilitators of online sessions for individuals with NDD noted that they found themselves less concerned about earlier-noted issues such as, for youth with autism, incidental concerns related to the lack of eye contact or unique hand gestures. Participants stated that such features of presentation had been viewed more problematically within in-person encounters, thus with more judgment and negative comment than was the case in online engagement. One participant described the receptivity of children/youth in speech language therapy in a virtual context, concluding,

With our speech language therapy, they have been able to work virtually with their families, and they have found that kids are much more relaxed and ready to do work, … and as successful, if not more successful. They didn’t feel that same awkwardness with some of those kids, and they really felt such a *buy in* from them… They even surprised their parents.

Some families were observed by participants to have become more connected and involved with their child’s programming than they were before the pandemic. Virtual connection also had allowed people to connect outside of the formal program setting and allowed for different types of experiences such as peer engagement and virtual attendance at unique places such as museums or zoos.

While beneficial for many, it is important to note that individuals in this sample with limited resources, less commonly had access to computers, the internet or phones. Such differentiated opportunities and access reportedly rendered it difficult for individuals and families with fewer socio-economic resources or who were most isolated to access services via technology or to follow online updates about pandemic guidelines.

## Discussion

**Fig. 1:**
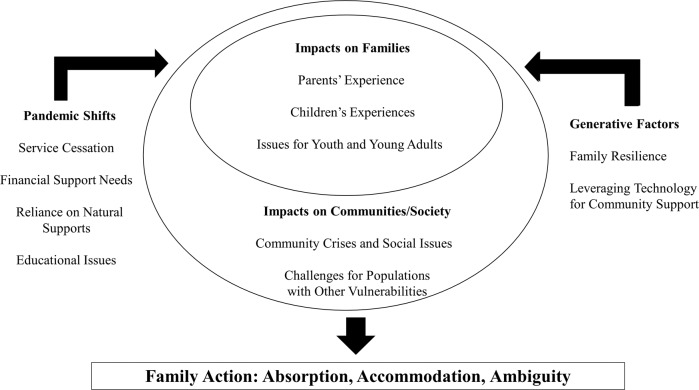
Shifts, impacts and generative responses

These findings highlight a range of experiences and impacts of the COVID-19 pandemic, largely reflecting challenges for individuals with NDD and their families. Yet, results also convey generative factors reflective of individual and family resilience in the face of adversity and significant system constraint. In considering impacts, it is important to recognize that the pandemic is not a static event, but rather a changing phenomenon over time. As summarized in Fig. 1, these findings amplify shifts and struggles such as service cessation or recalibration, the need for financial supports, the requisite of increased natural supports, if available, and school-related challenges. The impacts of the pandemic indeed have been substantial for individual families, with yet unknown outcomes both over the course of the pandemic (which continues at the time of writing) and beyond the pandemic, including possible lingering or long-term effects (e.g., mental health issues, unmet health needs). Gaps in services have been deeply felt by this population, revealing amplified risk for service delays in an already strained system of support, resulting in individual/family setbacks and morbidity such as mental health challenges, individual/family poverty, housing insufficiency, food insecurity, etc.

Yet amidst substantial losses and system gaps imposed and or exacerbated by the pandemic, generative or mitigating factors have surfaced, such as individual family resilience and technology utilization – each of which has eased some negative impacts of the pandemic on individuals with NDD and their families and in so doing, have nurtured adaptation and coping. While it is important to recognize the benefits of technology for some, others continue to face various accessibility issues, and gaps emerge in pivots to technology-based services (Mauldin et al., 2020). There are concerns of a ‘digital divide’ related to existing inequalities, such as students in at-home learning setups that lack stable internet connection, family caregivers who are unable to support their learning (Calarco, 2020), and/or remote communities without internet connectivity. Overall, the pandemic has differentially and deleteriously affected the population; the net result being experiences of *absorption, accommodation*, and *ambiguity*, as described below

First, given system gaps in care due to pandemic restrictions, individuals and families by default have directly *absorbed* system gaps and incorporated solutions as best as they can, by assuming greater responsibility for specialized NDD care and/or enduring without such care or support. This responsibility for care largely has been imposed on family caregivers, irrespective of its personal cost (Nicholas et al, 2020b).

Second, individuals have *accommodated* the continually shifting restrictions of the pandemic, requiring adjustment to imposed guidelines (e.g., physical distancing, restriction intensity/easing, mandated use of PPE, closing/opening/changing services, travel restrictions). They have experienced pandemic impositions and struggles over a lengthy duration (e.g., initial crisis, maintaining stamina amidst exhaustion and discouragement, functioning as restrictions ensue and numerous waves of the pandemic continue).

Third, individuals and families have experienced extreme *ambiguity* as they, like others globally, have endured continuing uncertainty, malaise, hope, discouragement, etc. related to the trajectory of the pandemic. What is unique among these families, however, is the interface of vulnerability and unmet need. The uncertainty about *how* and *to what extent* the disruptions and delays of assessment and service access will ultimately impact individuals with NDD, remains unknown. Concerns abound that assessment and service gaps will impose developmental impact in the short- and mid-term, but possibly also even generationally.

These common and unique struggles collectively call for rapid attention to strategies and services to proactively address gaps in the hope of limiting potential losses and negative impacts associated with the pandemic.

The pandemic has amplified system vulnerabilities such that individuals with NDD and their families are left underserved. Multiple causes for system disruption have been identified. For instance, NDD services have largely been deemed *non-essential*, justifying substantial reductions, closures and delays, with apparently limited consideration for the deleterious impacts on this population. Diagnostic, developmental and mental health supports, among other resources, have thus been delayed or otherwise rendered dispensable, with growing concern that short and long-term loss and deepening debilitation will result. As noted by participants, the short and longer-term or even generational impacts of these gaps have yet to be ascertained, and little has been documented about the potential and unknown risks of denying or delaying services for a population for whom intervention is integral to development and well-being.

Deleterious impacts of the pandemic are further noted in NDD cohorts living in congregate housing. In group homes and other congregate care settings, risk for COVID-19 has been demonstrated as amplified (CDC, 2020), with questions raised about PPE utilization/compliance and the lack of sufficient care and the lack of broader community engagement. The current lack of attention to risks imposed on disabled people in congregant housing invites critical scrutiny, including attention to co-existing health and mental health issues, care insufficiency, safety concerns and structural inequity. Identification of disability services as “non-essential” in the pandemic, seemingly highlights a devaluation of the service needs of disabled people in neoliberal society (Goodley & Lawthom, 2019; Kelly, 2016; Mitchell & Snyder, 2015), rendering disabled people marginalized in and potentially beyond the pandemic.

In the context of pandemic experiences of disabled individuals and their families, this population is notably under-served in a constrained system of health and social care – a position that has been markedly highlighted and seemingly amplified by the pandemic. Through many instances, as conveyed herein, if a given disabled individual had not been extensively supported by a family member, that individual would be at substantial risk for deleterious outcomes.

Yet, it seems unjust, unethical and inexcusable for family members to be left to fill glaring societal service gaps. Such issues further have gender and role implications, given the disproportionate role of women as family caregivers (Moyser & Burlock, 2018; Statistics Canada, 2020b), and the heightened impacts of the pandemic on marginalized groups (Canadian Human Rights Commission, 2020; Kantamneni, 2020). Greater resources for bolstering state-supported NDD care in disability services, family support, mental health, residential care, education and community housing, are urgently needed. Moreover, flexibility in service access and funding during crises such as a pandemic is paramount. The need for clarity about what and how services can be accessed, calls for more navigational supports for individuals with NDD and their families, particularly amidst the confusion and struggle of a pandemic. Sadly, services have diminished substantially in this pandemic just as needs have continued and indeed escalated. We recommend mental health and tangible supports for individuals and families as they walk through the uncertainties and struggles of navigating NDD in a pandemic.

These findings are a part of a larger *call for action*. This call is not new; however, these findings may be novel in amplifying the particular vulnerability and amplified risk for individuals with NDD in the face of a pandemic. As do previous studies, these findings point to the need for integrated responses at micro (e.g., support for the individual and family), mezzo (e.g., program development and integrated support and navigation), and macro (e.g., consistent information, policy devoted to the needs of individuals with NDD) levels. In advancing practice, programming and policy, recommendations invite greater access to care, bolstered communication with individuals and families during the pandemic, co-operation across services, and coordination across care sectors including health, education and social care to better meet the needs of individuals with NDD and their families. As a potential paradoxical ‘silver lining’, perhaps the pandemic has more brightly shone a light on deep societal issues and resource needs for people with NDD, in sounding an alarm for action and change.

## Limitations and Research Recommendations

While the study has offered insights from service providers, broader representation across the range of salient stakeholders is warranted. We especially advocate future research that elicits the first-hand experiences and perceptions of disabled individuals and their family members, and in that regard, we acknowledge that perceptions herein must be recognized as filtered through the perspectives of service providers. We accepted this potential limitation as a trade-off for the benefit of accessing, in our sample, diverse jurisdictional representation that included representation of many individuals and families, based on caseloads of service providers from urban and rural jurisdictions. We further opted for this approach and sample due to our sense of urgency in accessing data and sharing findings with policy, program planning and practice leaders, based on our pragmatic commitment to rapidly generate wide-reaching pandemic-related experiences and recommendations for practical utility. In moving forward, we recommend longitudinal research to ascertain the nature, extent and duration of pandemic effects over the mid and long term, including granular analysis of processes that mediate outcomes.

To our knowledge, this research is one of a relatively small volume of published studies to date addressing NDD in the context of the COVID-19 pandemic. Such research is urgently needed as the adversity imposed by system shifts and protracted service gaps has been profoundly felt. In moving forward, critical reflection and intentional proactive action are needed.
